# Supplementary material for: Multi‐dataset identification of innovative feature genes and molecular mechanisms in keratoconus
Source: J Cell Mol Med. 2024 Sep 19;28(18):e70079. doi: 10.1111/jcmm.70079 (PMC11412914; doi:10.1111/jcmm.70079)
Supplement: Supplementary file 2 — Material S2. [file JCMM-28-e70079-s002.zip › 7.GSEA analysis/4.MYRF/KEGG_PROTEIN_EXPORT.html]

Details for gene set KEGG\_PROTEIN\_EXPORT[GSEA]

|  || Dataset | MYRF.group.cls#h\_versus\_l |
| Phenotype | group.cls#h\_versus\_l |
| Upregulated in class | h |
| GeneSet | KEGG\_PROTEIN\_EXPORT |
| Enrichment Score (ES) | 0.2732301 |
| Normalized Enrichment Score (NES) | 0.87487334 |
| Nominal p-value | 0.6991701 |
| FDR q-value | 1.0 |
| FWER p-Value | 1.0 |
Table: GSEA Results Summary

  

Fig 1: Enrichment plot: KEGG\_PROTEIN\_EXPORT      
 Profile of the Running ES Score & Positions of GeneSet Members on the Rank Ordered List

  

| SYMBOL | TITLE | RANK IN GENE LIST | RANK METRIC SCORE | RUNNING ES | CORE ENRICHMENT || 1 | SEC61A2 | na | 5816 | 0.198 | 0.0366 | Yes |
| 2 | SRP54 | na | 10471 | 0.087 | -0.0078 | Yes |
| 3 | SRP9 | na | 12482 | 0.057 | -0.0072 | Yes |
| 4 | SRP19 | na | 12695 | 0.054 | 0.0423 | Yes |
| 5 | IMMP2L | na | 14120 | 0.037 | 0.0389 | Yes |
| 6 | SEC62 | na | 15650 | 0.021 | 0.0169 | Yes |
| 7 | SRP68 | na | 16016 | 0.018 | 0.0244 | Yes |
| 8 | IMMP1L | na | 16829 | 0.010 | 0.0113 | Yes |
| 9 | SRPRB | na | 16948 | 0.009 | 0.0173 | Yes |
| 10 | SEC11A | na | 17992 | -0.000 | -0.0124 | Yes |
| 11 | SEC11C | na | 18034 | -0.001 | -0.0127 | Yes |
| 12 | SPCS1 | na | 18224 | -0.003 | -0.0154 | Yes |
| 13 | SRP14 | na | 18841 | -0.009 | -0.0243 | Yes |
| 14 | SEC61B | na | 19167 | -0.012 | -0.0209 | Yes |
| 15 | SPCS2 | na | 20231 | -0.025 | -0.0262 | Yes |
| 16 | SEC63 | na | 20487 | -0.027 | -0.0053 | Yes |
| 17 | SRP72 | na | 22439 | -0.051 | -0.0086 | Yes |
| 18 | HSPA5 | na | 22668 | -0.055 | 0.0411 | Yes |
| 19 | SPCS3 | na | 23313 | -0.064 | 0.0888 | Yes |
| 20 | SEC61A1 | na | 23353 | -0.065 | 0.1545 | Yes |
| 21 | SEC61G | na | 23670 | -0.070 | 0.2179 | Yes |
| 22 | OXA1L | na | 25245 | -0.098 | 0.2732 | Yes |
Table: GSEA details [plain text format]

  

Fig 2: KEGG\_PROTEIN\_EXPORT      
 Blue-Pink O' Gram in the Space of the Analyzed GeneSet

  

Fig 3: KEGG\_PROTEIN\_EXPORT: Random ES distribution      
 Gene set null distribution of ES for **KEGG\_PROTEIN\_EXPORT**
